# Biomarkers of Berry Intake: Systematic Review Update

**DOI:** 10.1021/acs.jafc.3c01142

**Published:** 2023-07-27

**Authors:** Hamza Mostafa, Alex Cheok, Tomás Meroño, Cristina Andres-Lacueva, Ana Rodriguez-Mateos

**Affiliations:** † Biomarkers and Nutrimetabolomics Laboratory, Department of Nutrition, Food Sciences and Gastronomy, Nutrition and Food Safety Research Institute (INSA), Facultat de Farmàcia i Ciències de l’Alimentació, Universitat de Barcelona (UB), 08028 Barcelona, Spain; ‡ Centro de Investigación Biomédica en Red de Fragilidad y Envejecimiento Saludable (CIBERFES), Instituto de Salud Carlos III, Madrid 28029, Spain; § Department of Nutritional Sciences, School of Life Course and Population Sciences, Faculty of Life Sciences and Medicine, King’s College London, 150 Stamford Street, SE1 9NH London, U.K.

**Keywords:** Berries, Blueberry, Cranberry, Raspberry, Strawberry, Blackberry, Blackcurrant, BFIs

## Abstract

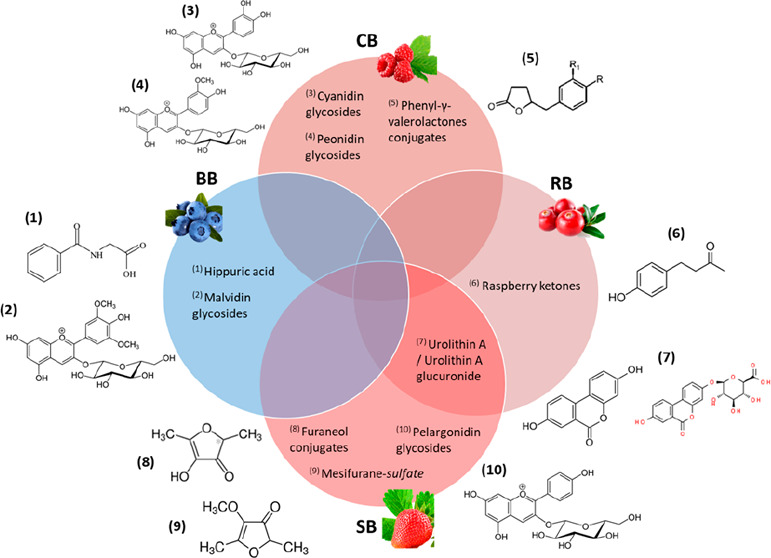

Berries are rich in (poly)phenols, and these compounds may be beneficial to human health. Estimating berry consumption through self-reported questionnaires has been challenging due to compliance issues and a lack of precision. Estimation via food-derived biomarkers in biofluids was proposed as a complementary alternative. We aimed to review and update the existing evidence on biomarkers of intake for six different types of berries. A systematic literature search was performed to update a previous systematic review on PubMed, Web of Science, and Scopus from January 2020 until December 2022. Out of 42 papers, only 18 studies were eligible. A multimetabolite panel is suggested for blueberry and cranberry intake. Proposed biomarkers for blueberries include hippuric acid and malvidin glycosides. For cranberries, suggested biomarkers are glycosides of peonidin and cyanidin together with sulfate and glucuronide conjugates of phenyl-γ-valerolactone derivatives. No new metabolite candidates have been found for raspberries, strawberries, blackcurrants, and blackberries. Further studies are encouraged to validate these multimetabolite panels for improving the estimation of berry consumption.

## 1 Introduction

Berries are fruits that are common in healthy dietary patterns as they are rich in phytochemicals, fiber, and micronutrients.^1,2^ Berries may play an important role in the prevention and treatment of chronic diseases such as cardiovascular diseases (CVD), cancer, diabetes mellitus (DM), and age-related cognitive decline according to an increasing number of randomized controlled trials (RCTs) and epidemiological studies.^3−5^ However, to understand the relationship between berries and health, it is essential to be able to quantify their consumption accurately. The development of novel dietary assessment methods to estimate food consumption is a current focus of nutrition research. Recent studies aim to discover and validate biomarkers of food intake (BFIs) in biofluids, typically in the blood and urine. BFIs can be effectively used to (i) validate dietary intake questionnaires, (ii) study physiological and pathological responses to food and food components, (iii) provide information on interindividual variability in food responses, and (iv) help to formulate personalized dietary recommendations.^6−8^ However, only a very limited number of biomarkers for specific foods have been proposed. Currently, there are no validated biomarkers for berries.

The most abundant phytochemicals in berries are (poly)phenols, in particular anthocyanins, ellagitannins, flavonols, flavan-3-ols, and phenolic acids.^9^ A large number of circulating metabolites, including phase II and gut microbial metabolites derived from these compounds have been reported after the consumption of different types of berries in human intervention studies.^10−12^ Previous studies have proposed ellagitannins and their gut microbial metabolites, urolithins (Uros), as biomarkers of strawberry and raspberry intake;^13,14^ aromatic compounds like furaneol as biomarkers of strawberry intake;^15^ and anthocyanins (ACN) like malvidin and its derivatives as biomarkers of blueberry intake.^16,17^ However, these metabolites are ubiquitous and can be found in many types of foods such as nuts, red grapes, and red wine. Therefore, they cannot be used as single biomarkers due to the lack of specificity coupled with their abundance in a habitual diet. Similarly, phenolic acids are present in berries and they are also formed in the colon via the gut microbial metabolism of anthocyanins and other berry (poly)phenols; however, they are also abundant in many foods, such as coffee, tea, bread, and other fruits and vegetables. A potential solution to this is to use multibiomarker panels, which have been previously proposed to be more advantageous than the single biomarker approach.^18^ In the past few years, a number of studies have provided additional information on the berry metabolome; therefore, in this work, we aimed to update a previous systematic review of biomarkers of berry intake^19^ to capture additional candidates beside the previously proposed metabolite panels.

## 2 Methodology

**Figure 1:**
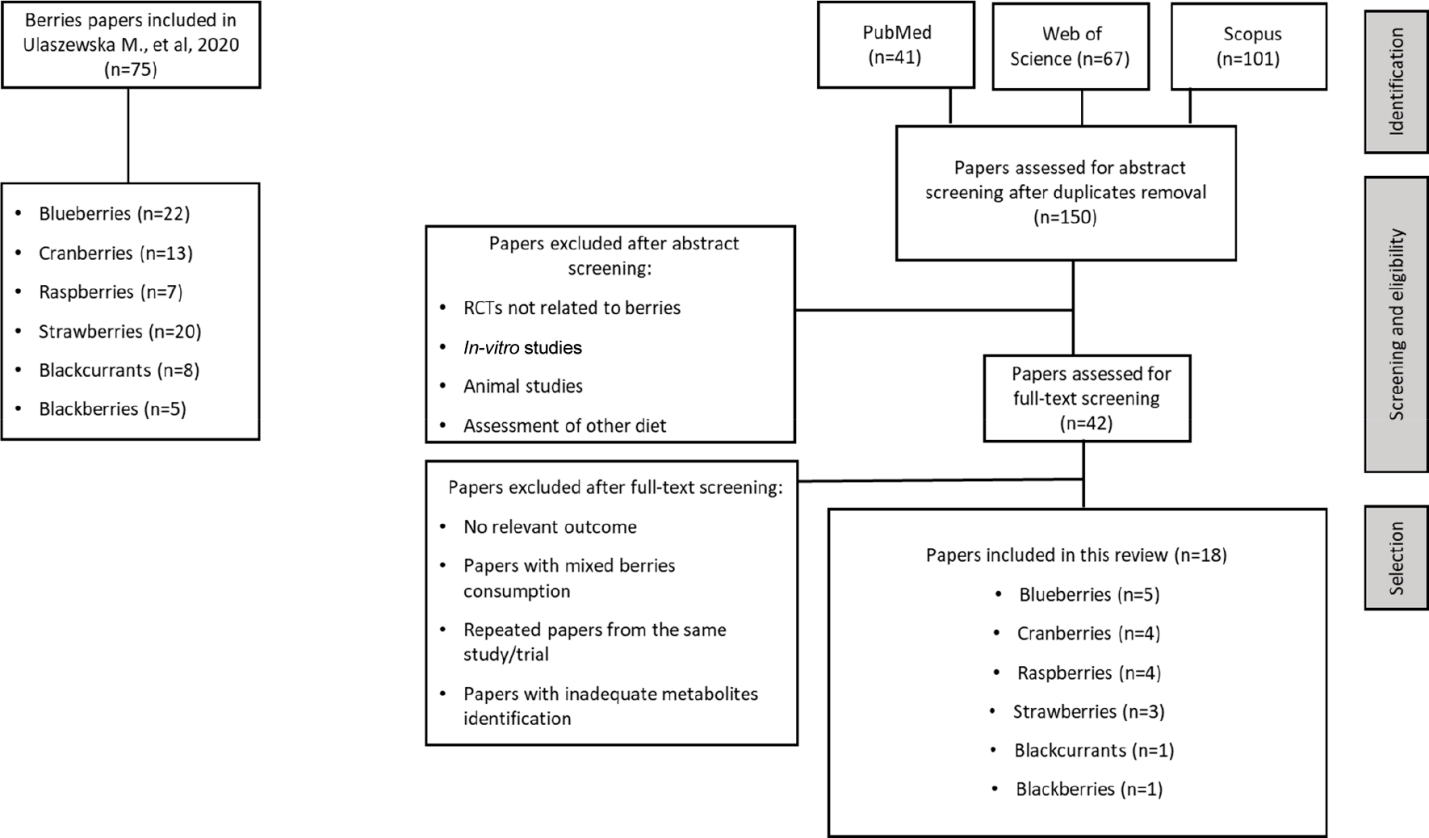
Study selection bibliography search flow diagram.

We conducted the current systematic review according to the PRISMA statement (Preferred Reporting Items for Systematic Reviews and Meta-Analysis) as detailed in Supplementary Table 1. Our review employed an extensive search using the following keywords (Fruit name* OR botanical name), AND (urine or plasma or serum or excretion or blood) AND (human* OR men OR women OR patient* OR volunteer* OR participant*) AND (biomarker* OR marker* OR metabolite* OR biokinetics OR biotransformation OR pharmacokinetics OR bioavailability OR ADME) AND (intake OR meal OR diet OR ingestion OR administration OR consumption OR eating OR drink) across three electronic databases: PubMed, Web of Science, and Scopus. The previous search covered published literature until December 2019. To extend upon that, our systematic search included trials published in English only that were conducted only on berries between January 2020 and December 2022. Short- and long-term human intervention studies conducted on berry intake were selected for this review. As detailed in Figure 1, the main exclusion criteria were animal and *in vitro* studies and papers assessing irrelevant diets. Two investigators independently performed the study selection and data extraction.

## 3 Results and Discussion

We found 41 papers in PubMed, 67 in Web of Science, and 101 in Scopus. Duplicated papers or papers found to be irrelevant to our objective were excluded from the review. From there, only 42 articles fulfilled the inclusion and exclusion criteria of this review. After full-text screening, 18 papers were selected and analyzed, as detailed in Figure 1 and Table 1.

**Table 1:** List of the Studies Included for the Assessment of Biomarkers of Berry Intake^a^

Reference	Intervention	Study characteristics	Analytical method	Candidate biomarkers (blood)	Candidate biomarkers (urine)
Blueberries (BB)					
(39)	50g freeze-dried powder.	Double-blind parallel RCT	Targeted metabolomics LC-MS/MS analysis	Increased	
	Phytochemical content not reported	Duration: 8 w	Plasma samples (w0 and w8)	- HA	
		49 participants	6 identified metabolites	Decreased	
		- At risk of MetS		- Ornithine	
		- 55% females		- Hypoxanthine	
		- Age: 22–53 y		- Diacylglycerol	
		Compliance: 92.7%		- Indoxyl sulfate	
				- Ceramide	
(40)	26 g freeze-dried powder containing 1243 mg of total (poly)phenols	Double-blind parallel RCT	UPLC-MS/MS analysis	- 2,6-dimeO-phenol	**-** Phloroglucinaldehyde
		Duration: Acute single dose	Serum samples (30, 60, 90, 120, 180, 360 min and 24 h), Urine samples (24 h)	- 3-(4-OH-3- meO-ph)-PrA	- 3-(3,4-diOH-ph)-PrA
		45 participants	21 identified metabolites	- 3-OH-4-meO-PA	- 3-OH-HA
		- With MS		- 3-meO-PA-4-*S*	- Syringic acid
		- 64% males		- 4-OH-HA	- 4-OH-BA
		- Age: 63.4 ± 7.4 y		- HA	- Isovanillic acid-gluc
				- HA-*S*	- Benzoylglutamic acid
				- OH-meO-BA-*S*	- 3-Caffeoylquinic acid
				- 3-(3-meO-ph)-PrA	- HA
				- Methoxy-PA-gluc	- BA-4-S
					- 3,4-OH-BA-3/4-*S*
					- DOPAC
(41)	24g freeze-dried powder containing 36 mg/g of total (poly)phenols	Double-blind parallel RCT	UPLC-QQQ-MS analysis	- HA	
		Duration: 90 d	Plasma samples (0, 2 h)	- Phloroglucinaldehyde	
		38 participants	(d0, d45, d90)	- Syringic acid	
		- Healthy	11 identified metabolites	- Ferulic acid-gluc	
		- 68% females		- Cya-3-galac	
		- Age: 60–75 y		- Cya-3-glu	
		Compliance: 99.2%		- Mal-3-galac	
				- Mal-3-glu	
				- Peo-3-xyl	
				- Peo-3-gluc	
				- Pet-3-glu	
(42)	25 g of (*Vaccinium myrtillus* (VM))	Single-blind parallel randomized trial	Untargeted metabolomics LC-HRMS analysis	After VM consumption:	
	25 g of (Vaccinium Corymbosum (VC)) supplements in a capsule containing 39 mg of catechin equivalent/g (total (poly)phenols∼ 1 g)	Duration: Acute single dose	Serum samples (30, 60, 120, 240, and 360 min)	- Uric acid	
		20 participants	12 identified metabolites	- Inosine	
		- Healthy		- α-Hydroxyhippuric acid	
		- 1 male, 9 females		- Catechol-*S*	
		- Age: 25–60 y		- me-catechol-*S*	
				- Abscisic acid glucuronide	
				- Azelaic acid	
				- OH-ph-PrA-*S*	
				After VC consumption:	
				- Caprylic acid (hydroxyl octanone)	
				- Tetrahydro-me-ß-carboline dicarboxylic	
				- Octahydro-methyl-ß-carboline dicarboxylic	
				- Citric acid for both VM and VC.	
(43)	13.3 g blueberry juice daily, containing 766 mg of total (poly)phenols	Single-blind between-groups trial	UHPLC-Q-TOF-MS analysis		- HA
		Duration: 4 w	Urine samples (24 h)		- Dihydrocaffeic acid-3-*S*
		15 participants	2 identified metabolites		
		- Healthy			
		- 7 males, 8 females			
		- Age: 7–10 y			
Cranberries (CB)					
(10)	Cranberry juice containing 375, 716, 1131, 1396, 1741 mg of total flavan-3-ols	Double-blind six-arm crossover RCT	Targeted UHPLC-ESI-QqQ-MS/MS analysis	- 5-(diOH-ph)-γ-VL-gluc (3′,4′,5′)	- 5-(5′–OH-ph)-γ-VL-3′-gluc
		Duration: Acute single dose	Plasma samples (0, 1, 2, 4, 6, 8 and 24 h), Urine samples (0, < 8, 8–24 h)	- 5-(5′- OH-ph)-γ-VL-3′-gluc	- 5-ph-γ-VL-4′-gluc
		10 participants	22 identified metabolites	- 5-(3′,5′-diOH-ph)-γ-VL	- 5-(3′–OH-ph)-γ-VL-4′-gluc
		- Healthy		- 5-(diOH-ph)-γ-VL-*S* (3′,4′,5′)	- 5-ph-γ-VL-*S*-gluc isomer (3′,4′)
		- Males		- 5-ph-γ-VL-4′-gluc	- 5-(4′–OH-ph)-γ-VL-3′-gluc
		- Age: 18–35 y		- 5-(3′–OH-ph)-γ-VL-4′-gluc	- 5-(3′,4′-diOH-ph)-γ-VL
				- 5-ph-γ-VL-*S*-gluc isomer (3′,4′)	- 5-(5′–OH-ph)-γ-VL-3′-*S*
				- 5-(4′–OH-ph)-γ-VL-3′-gluc	- 5-ph-γ-VL-meO-gluc isomer (3′/4′)
				- 4-OH-5-(OH-ph)-VA-*S* (3′/4′) isomer 1	- 5-ph-γ-VL-3′-gluc
				- 4-Hydroxy-5-(OH-ph)-VA-gluc (3′/4′)	- 5-(OH-ph)-γ-VL-*S* (3′,4′ isomers)
				- 5-(5′–OH-ph)-γ-VL-3′-*S*	- 5-ph-γ-VL-meO-*S* (3′,4′) isomer 1
				- 5-ph-γ-VL-meO-gluc isomer (3′/4′)	- 5-ph-γ-VL-3′-*S*
				- 5-OH-ph -γ-VL-meO-gluc (3′,4′,5′)	- 5-ph-γ-VL-meO-*S* (3′,4′) isomer 2
				- 5-ph-γ-VL-3′-gluc	
				- 4-OH-5-(OH-ph)-VA-*S* (3′/4′) isomer 2	
				- 5-(OH-ph)-γ-VL-meO-*S* (3′,4′,5′)	
				- 5-(OH-ph)-γ-VL-*S* (3′,4′ isomers)	
				- 5-ph-γ-VL-4′-*S*	
				- 5-ph-γ-VL-meO-*S* (3′,4′) isomer 1	
				- 5-ph-γ-VL-3′-*S*	
				- 5-ph-γ-VL-meO-*S* (3′,4′) isomer 2	
(64)	9 g whole cranberry freeze-dried powder containing 525 mg of total (poly)phenols	Double-blind parallel RCT	Targeted quantitative UPLC-MS analysis	- 1 Flavonol	- 5 Flavonols
		Duration: 1 month	Plasma samples (0, 2 h) (d1, 1 month), Urine samples (24 h) (d1, 1 month)	- 2 Benzene diols and triols	- 7 Benzene diols and triols
		45 participants	130 identified metabolites	- 2 BAL	- 3 BAL
		- Healthy		- 5 HAs	- 5 HAs
		- Males		- 11 BAs	- 14 BAs
		- Age: 25 ± 3 y		- 12 CAs	- 15 CAs
				- 6 PAs	- 6 PAs
				- 12 ph-PrAs	- 11 ph-PrAs
				- 5 ph-γ-VLs and ph-VAs	- 8 ph-γ-VLs and ph-VAs
				Increased after 2 h:	Increased after 1 day:
				13 in plasma	13 in urine
				Increased after 1 month:	Increased after 1 month:
				4 in plasma	13 in urine
(65)	6 bottles/3 d (250 mL per bottle, twice a day) of 54% cranberry juice containing 913 ± 7 mg of total (poly)phenols Mean ± SD	Crossover RCT	Untargeted UHPLC-Q-orbitrap-HRMS-based metabolomics analysis		- Quinic acid
		Duration: 3 d	Spot urine samples (d0, after d3)		- Coumaric acid
		15 participants	16 identified metabolites		- 4-OH-5-(OH-ph)-VA-*S*
		- Healthy			- 5-(diOH-ph)-γ-VL-*S*
		- Females			- Diphenol-gluc
		- Age: 21–29 y			- 3,4-diOh-ph-PA
					- 3-(OH-ph)-PA
					- 4-me-gallic acid,
					- triOH-BA
					- 1,3,5-trimeO-benzene
					- Homocitric acid
					- HA
					- 3-OH-3-carboxy-me-adipic acid
					- (2)3-isopropylmalate
					- Pimelic acid
					- N-acetyl-l-glutamate 5-semialdehyde
(66)	6 bottles/21 d (250 mL per bottle, twice a day) of 54% cranberry juice	Double-blind crossover RCT	UPLC-MS untargeted metabolomics analysis	- Quinic acid ^⧺^	
		Duration: 21 d		- 3-(OH-ph)-PA ^⧺^	
		16 participants		- (*S*)-Homostachydrine	
		- Healthy		- et-(methylthio)methyl disulfide	
		- Females		- Catechol-*S*^⧺^	
		- Age: 21–29 y		- Vanilloloside ^⧺^	
		Compliance: 100%		- *S*-acetyl dihydroasparagusic acid	
				- Pyrocatechol	
				- Guaiacol	
				- HA ^⧺^	
				- Glycerol 3-phosphate	
				- diOH-quinoline ^⧺^	
				- OH-pyruvic acid	
				- 3,4-diOH-ph-glycol	
				- Guanidoacetic acid	
				- 2-Chloromaleylacetate	
				- 2-Phenylacetamide	
				- Tyrosine ^⧺^	
				- 3-Isopropylmalate	
				- 2-Chloromaleylacetate	
				- Lanthionine ketimine	
				- Prolyl-Hydroxyproline	
				- Tyramine-*O*-*S*	
	containing 913 ± 7 mg of total (poly)phenols Mean ± SD		Plasma samples (d0, d3, d21)	- (3,4,5,6-tetrahydroxyoxan-2-yl)me-4-OH-benzoate	
			25 identified metabolites	- {4-[2,3-dioxo-3-(2,4,6-trihydroxy-3-meO-ph)propyl]-2-OH-6-meO-ph}oxidanesulfonic acid	
				^⧺^ Identified in a previous study^111^	
Raspberries (RB)					
(77)	- 250 g frozen raspberries containing 203.6 ± 11.1 mg/meal of total (poly)phenols Mean ± SD	Single-blind three-arm crossover RCT	UHPLC-QTOF analysis	- Cya-3-sop	
		Duration: Acute single dose	Plasma samples (0, 0.5, 1, 2, 4, 6, 7, 8, and 24 h)	- Cya-3-glu	
		32 participants	24 identified metabolites	- Cya glucosylrutinoside	
		- 12 preDM-IR and 11 healthy		- Cya-3-rut	
		- 15 males, 17 females		- Pel-3-sop	
		- Age: 34 ± 12		- Pel-3-glu	
				- me-cya-sop	
				- me-cya-3-glu	
				- 8-OH-Uro-3-gluc	
				- Uro-3-gluc	
				- 4′–OH-CA	
				- OH-CA	
				- CA-gluc	
				- 4′–OH-3′-meO-CA	
				- OH-meO-CA	
				- meO-CA-gluc isomer 1	
				- etO-CA-gluc isomer 2	
				- 2,4,6-triOH-BAL	
				- 3,4-diOH-BA	
				- 2,3- diOH-BA	
				- 4-OH-PA	
				- diOH-ph-PA isomer	
				- HA	
	- 125 g frozen raspberries containing 101.8 ± 5.6 mg/meal of total (poly)phenols Mean ± SD			- HA-gluc	
(78)	Frozen raspberry drink containing 388.4 ± 3.3 mg of total (poly)phenols Mean ± SD	Single-blind crossover RCT	UHPLC-QQQ analysis	- 13 ACN derivatives	Increased:
		Duration: 4 w	Plasma samples (0, 0.5, 1, 2, 3, 4, 24h), Urine samples (0, 1,2,3,4, 24h)	- 7 Uros derivatives	- Total Uros
		35 participants:	123 identified (poly)phenolic metabolites	- 9 ph-γ-VLs derivatives	- Total ph-γ-VL
		- 25 preDM-IR and 10 healthy		- 94 Phenolic acid derivatives:	
		- 17 males, 18 females		- 10 BALs	
		- Age: 34 ± 3		- 24 CAs	
				- 19 ph-PrA	
				- 17 PAs	
				- 16 BAs	
				- 8 HAs	
				Increased:	
				- Total Uros	
				- Total ph-γ-VL	
				- Select PA(CAs, ph-PrAs and HAs)	
				Decreased:	
				- Total ACN	
(79)	280 g frozen raspberries	Two-arm parallel RCT	Targeted metabolomics analysis using MxP Quant 500 kit	- Cholesterol 1-pentadecanoate	
	No phytochemical content reported	Duration: 8 w	Plasma samples (w0, w4, w8)	- 1-Palmitoyl-2-palmitoyl-3-docosahexaenoyl-glycerol	
		48 participants	10 identified metabolites	- 1-Octadecanoyl-2-(9Z-hexadecenoyl)-3-(9Z-tetradecenoyl)-glycerol	
		- Obese		- 1-Palmitoleoyl-2-palmitoleoyl-3-linoleoyl-glycerol	
		- 16 males, 32 females		- 1-Arachidonyl-2-docosapentaenoyl-*sn*-glycero-3-phosphocholine	
		- Age: 18–60 y		- 1-Octadecanoyl-2-octadecanoyl-3-(9Z-tetradecenoyl)-glycerol	
		Compliance: 92.8%		- β-Alanine	
				- Trimethylamine N-oxide	
				- Deoxycholic acid glycine conjugate	
				- Hexosylceramide	
(80)	- 10 g or 20 g Raspberry nectar (containing 17 mg and 46 mg of ETs respectively)	Five-arm parallel RCT	UPLC/MS/MS analysis	- Uro A	- Uro A
	- 10 g or 20 g Raspberry confection (containing 25 mg and 50 mg of ETs respectively)	Duration: 4 w	Plasma samples, Urine (spot and 24 h)	- Uro C	- Uro B
		40 participants	5 identified metabolites		- Uro C
		- Healthy			- Uro D
		- Male			- dime-ellagic acid
		- Age: 60.2 ± 7 y			
		Compliance: 100%			
Strawberries (SB)					
(84)	50 g strawberry freeze-dried powder containing 450.7 mg of total (poly)phenols	Double-blind crossover RCT	UPLC-QQQ analysis	Decreased:	
		Duration: 4 w	Plasma (w0, w4)	**-** Secondary BAs	
		34 participants	37 identified bile acids (BA) species	- Deoxycholic acid	
		- Obese		- Lithocholic acid (and their glycine conjugates)	
		- 17 males, 17 females		- Glycoursodeoxycholic acid	
		- Age: 52.6 ± 7.1 y			
(82)	50g whole strawberry freeze-dried powder containing 450.7 mg of total (poly)phenols	Double-blind crossover RCT	Targeted metabolomics UHPLC-ESI-MS/MS analysis	8 increased:	
		Duration: 4 w	Plasma samples (0, 1 h)	- 3-meO-BA-4-S	
		34 participants	17 identified phenolic metabolites	- 3-meO-PA	
		- With MHC		- 3-OH-ph-γ-VL-4-*S*	
		- 17 males, 17 females		- 4-OH-PA	
		- Age: 53 ± 1 y		- 4-OH-3-meO-BA-me ester	
				- 3-(4-OH-ph)-PA-3-*S*	
				- OH-CA-*O*-gluc	
				- 4-OH-3,5-dimeO-PA	
				4 decreased:	
				- 4-meO-CA	
				- 3-me-HA	
				- OH-BAL-*O*-gluc	
				- 3-(4-meO-ph)-PA-3-gluc	
(83)	- 26 g strawberry freeze-dried powder + Beige diet daily	One-arm parallel trial	LCMS/MS analysis	- Pel-3-gluc	- Pel-3-gluc
	- Beige diet only	Duration: 4 w for (Beige diet + SB), 2 w for Beige diet only	Serum samples (w0, w4, w6), Urine samples (24 h) (w0, w4, w6)		- Uro A-gluc
	No phytochemical content reported	15 participants	4 identified metabolites		- dime-ellagic acid-gluc
		- Healthy			
		- 6 males, 8 females			
		- Age: 18–55 y			
Blackcurrants (BC)					
(95)	300 mg blackcurrant extract capsule coontains 105 mg of ACN	One-arm trial	Reversed-phase HPLC analysis	- Gallic acid	
		Duration: acute single dose	Plasma samples (0, 1, 1.5, 2, 3, 4, 5, 6 h)	- PCA	
		20 participants	2 identified metabolites		
		-Healthy			
		- 11 males, 9 females			
		- Age: 28 ± 7 y			
Blackberries (BLB)					
(103)	400 mL of commercial blackberry nectar containing 100 mg of Cya-3-glu	One-arm trial	LC-IMS-QTOF-MS and LC-LIT-MS analysis		- Two sulfated cya derivatives
		Duration: acute single dose	Urine samples (24 h)		- One sulfated cya-3-gluc
		Two participants	2 identified metabolites		
		- Healthy			
		- Age and sex: unreported			

a Data are presented as Mean ± SD. Study compliance only reported for long-term interventions when data was available. BB: blueberries, CB: cranberries, RB: raspberries, SB, strawberries, BC: blackcurrants, BLB: blackberries, VM: *Vaccinium myrtillus*, VC: vaccinium corymbosum, RCT: randomized controlled trial, preDM-IR: prediabetes and insulin resistance, MHC: moderate hypercholesterolemia, MetS: metabolic syndrome, HPLC: high pressure liquid chromatography, UPLC: ultra performance liquid chromatography, QQQ: triple quadrupole, MS: mass spectrometry, IMS: ion mobility separation, HRMS: high resolution mass spectrometry, LIT: linear ion trap, ESI: electrospray ionization, QTOF: quadrupole time-of-flight, HA: hippuric acid, 4-OH-BA: 4-hydroxybenzoic acid, DOPAC: 3,4-dihydroxyphenylacetic acid, BA: benzoic acid, CA: cinnamic acid, 4-OH-BA: 4-hydroxybenzoic acid, 3,4-OH-BA: 3,4-Hydroxybenzoic acid-3/4-sulfate*s*, BAL: benzaldehydes, Cya-3-galac: cyanidin-3-galactosdie, Cya-3-glu:cyanidin-3-glucoside, Mal-3-galac: malvidin-3-galactoside, Mal-3-glu: malvidin-3-glucoside, Peo-3-xyl: peonidin-3-xyloside, Peo-3-gluc: peonidin-3-glucoronide, Pet-3-glu: petunidin-3-glucoside, Pel-3-gluc: pelargonidin-3- glucuronide, OH-ph: hydroxyphenyl, diOH-ph: dihydroxyphenyl, ph: phenyl, VL: valerolactones, VA: valeric acid, PCA: protocatechuic acid, PrA: propionic acid, ph-PrA: phenylpropionic acid, PA: phenylacetic acid, 4-OH-PA: 4-hydroxyphenylacetic acid, me: methyl, dime: dimethyl, meO: methoxy, dimeO: dimethoxy, meO-ph: methoxyphenyl, et: ethyl, etO: ethoxy, ETs: ellagitannins, Uros: urolithins, Uro A: urolithin A, Uro B: urolithin B, Urol C: urolithin C, Uro D: urolithin D.

### 3.1 Description of the Selected Trials

Among the 18 studies, 8 studies had a berry drink as an intervention (in the form of juices, nectars, or frozen juices), 2 studies used extract capsules, 1 study used frozen berries, and 7 studies used freeze-dried powder dissolved in water. Twelve studies included only healthy participants. The age of the participants across all of the studies included in this review ranged from 21 to 75 years, except for one study, which was conducted on children (7–10 years old). Two studies had female participants only; while five studies had only male participants. There were 5 studies classified as acute interventions (single dose) while the long-term interventions were between 21 days and 90 days as shown in Table 1. All included studies performed analysis without enzymatic treatment with glucuronidase and sulfatase.

### 3.2 Biomarkers of Berry Intake

#### 3.2.1 Blueberries

A total of 22 studies investigating the metabolic fate of (poly)phenols after blueberry intake were included in the systematic review of Ulaszewska et al.^16,17,20−38^ They concluded that the major ACN found in blood and urine was delphinidin and malvidin glycosides. Various phenolic metabolites were also found after acute and chronic intake of blueberries such as benzoic, ferulic, catechol, hippuric, and phenyl valeric acids and phenyl-γ-valerolactone (ph-γ-VL) derivatives. However, these metabolites were not specific to blueberry intake and can also be produced after the intake of many other fruits and vegetables. Thus, there were no metabolites identified as BFIs for blueberry, and it was suggested that more trials were needed. In our systematic search, 5 additional studies that investigated plasma or urine metabolites after blueberry consumption were identified.^39−43^ Two of them were acute, single-dose studies, while the rest were long-term trials. One study administered a drink, while 4 studies used reconstituted freeze-dried powder. Only one study detected ACN in plasma namely cyanidin-3-galactoside (cya-3-galac), cyanidin-3-glucoside (cya-3-glu), malvidin-3-galactoside (mal-3-galac), peonidin-3-xyloside (peo-3-xyl), peonidin-3-glucuronide (peo-3-gluc), and petunidin-3-glucoside (pet-3-glu).^41^ Phenolic acid derivatives and small low molecular weight compounds were found in all 5 studies, including syringic acid, isovanillic acid, ferulic acid, benzoic acid, catechol, and their derivatives, 3-caffeoylquinic acid, hydroxyphenylpropionic acid sulfate (OH-ph-PrA-*S*), 2,6-dimethoxyphenol (2,6-dimeO-phenol), and 3,4-dihydroxyphenylacetic acid (3,4-diOH-PA) (Table 1). Hippuric acid was the only common metabolite found in all of the studies regardless of the type of biofluid, study design or trial duration. In Lapo Renai et al.,^42^ 12 metabolites were identified in serum using untargeted metabolomics after a single dose of *Vaccinium myrtillus* and *Vaccinium corymbosum* supplements (bilberry and blueberry, respectively), including some phenolic compounds, uric acid, inosine, and abscisic acid glucuronide (Table 1). Five of those metabolites were associated with blueberry consumption for the first time (citric acid, azelaic acid, caprylic acid, and two derivatives of *ß*-carboline dicarboxylic acid). However, the authors concluded, in agreement with previous data, that these metabolites are not specific to blueberry intake as they might be biomarkers of other fruits and vegetable consumption, as well as alcohol, coffee and fructose intake.^44−47^ Some endogenous metabolites such as inosine, uric acid, ornithine, and hypoxanthine were increased or decreased in blood samples after the intake of blueberry in several studies (Table 1). Nevertheless, endogenous metabolites could be affected by numerous intrinsic factors, hence disqualifying them as specific biomarkers for blueberry intake. In line with the result from the previous review, we suggest that the simultaneous presence of hippuric acid with malvidin glycosides could be an indicator of blueberry intake. This could potentially be used to assess compliance in human studies. However, they may not specific enough to be considered BFIs of blueberries within the overall diet of an individual, as there are many other anthocyanin-rich foods containing malvidin glycoside, and hippuric acid is a metabolite found after the consumption of many different types of (poly)phenols and also part of other endogenous pathways.^48,49^ In addition, intact anthocyanins are not an optimal biomarker of intake due to their instability and low concentration in biofluids.^50^

#### 3.2.2 Cranberries

Thirteen studies^51−63^ using untargeted and targeted metabolomic approaches in plasma and urine after cranberry consumption were included in Ulaszewska et al. systematic review,^19^ revealing that the most abundant ACN found were the arabinoside, glucoside and galactoside derivatives of peonidin and cyanidin. In addition, various phenolic metabolites were identified after cranberry intake such as sulfate conjugates of catechols, ferulic acid, coumaric acid, and ph-γ-VL derivatives alongside other metabolites such as hippuric acid, citramalic acid, and derivatives of terpenes and iridoids. Nevertheless, these metabolites are not specific BFIs for cranberry intake, as they are also metabolites formed after the consumption of many other (poly)phenol-rich foods. In this work, we identified 4 additional studies^10,64−66^ exploring the plasma and urine metabolome after cranberry consumption. Two studies^10,64^ used targeted analysis, whereas the other 2 used untargeted analysis.^65,66^ All of them are RCTs with interventions ranging from a single dose^10^ up to a 4 week daily consumption. Only one study administered reconstituted cranberry powder,^64^ while the rest used cranberry juices. Following up with previous work^58^ using untargeted metabolomics for cranberry intake biomarker discovery, Liu et al. have proposed newly identified exogenous discriminant metabolites, including 4-hydroxy-5-(hydroxyphenyl)-valeric acid sulfate, 5-(dihydroxy-ph)-γ-VL sulfate, pyrocatechol, guaiacol, (S)-homostachydrine, ethyl (methylthio)methyl disulfide, and *S*-acetyl dihydroasparagusic acid.^65,66^ However, these compounds have been found in biofluids after the consumption of many other foods,^67,68^ so unlikely to be good BFIs. Additional endogenous metabolites such as glycerol 3-phosphate, dihydroxyquinoline, and guanidoacetic acid were also found in this work (Table 1); however, they should not be considered as BFIs due to their susceptibility to changes by many other factors. Another study detected 56 and 74 (poly)phenols metabolites in plasma and urine respectively via a targeted approach^64^ after participants consumed 9 g of reconstituted cranberry powder daily for a month. Overall, 13 metabolites were found to have increased in plasma as well as 13 in urine samples. However, these are mainly derivatives of ph-γ-VLs, phenylvaleric acids (ph-VAs), cinnamic acids (CAs) and benzoic acids (BAs) which were previously excluded as BFIs in the previous systematic review, as these are metabolites of many other flavan-3-ol rich foods. Another study from the same team^10^ performed a targeted analysis of 22 ph-γ-VLs and ph-VAs in plasma and urine after an increasing dose (375–1741 mg) of total flavan-3-ols. The ph-γ-VLs in both plasma and urine showed a linear dose–response with the glucuronide and sulfate derivatives of 5-(3′,4′-dihydroxyph)-γ-VL being the most dominant metabolites. Although 5-(3′,4′-dihydroxyph)-γ-VL and some of its derivatives were previously ruled out as BFIs in Ulaszewska M. et al.,^19^ here the authors suggested that its sulfate and glucuronide conjugates could serve as a biomarker for the intake of cranberry flavan-3-ols in the context of controlled clinical trials, as they are not specific enough for being biomarkers of general cranberry intake. These gut microbial metabolites have been proposed as biomarkers of flavan-3-ol intake in previous studies, and have been successfully used in an epidemiological study investigating associations between flavan-3-ol intake and blood pressure.^69,70^ Taken together, these conjugates cannot be considered as specific BFIs of cranberries. Perhaps they could be used as indicators for intake when they appear alongside some ACN associated with cranberry intake, possibly as a collective multibiomarker panel. Further studies are warranted to confirm this.

#### 3.2.3 Raspberries

Seven studies^12,71−76^ investigating circulating raspberry metabolites were considered in the previous systematic review.^19^ Various ACN, phenolic, and ellagitannins metabolites were found in low concentrations in plasma and urine such as cya-3-glu, hippuric acid, ferulic acid, phenyl acetic acid, Uros and ellagic acid derivatives; however, these metabolites are not specific to raspberry consumption. Some animal models detected the formation of raspberry ketone 4-(phenylhydroxyphenyl)-2-butanone and its derivatives 4-(4-hydroxyphenyl)butan-2-ol and 4-OH-PA in urine and they have been proposed as BFIs for raspberries.^75^ These ketones can be rapidly absorbed from the gastrointestinal tract and are found exclusively in raspberries. To date, raspberry ketone metabolites have been detected in animal models but not in humans. Nevertheless, due to the widespread use of raspberry ketone as a food additive, it is not suitable to be used as single biomarkers for raspberry consumption. The use of raspberry ketones along with other raspberry-derived metabolites such as Uros has been proposed as a multi-BFIs for raspberry intake in the previous systematic review. The authors concluded that human studies are needed to validate these metabolites for use as BFIs of raspberries.^19^ Our systematic review captured 4 new publications investigating the raspberry metabolome.^77−80^ Two studies from the same research group^77,78^ collectively measured up to 123 (poly)phenolic metabolites in plasma and urine of individuals with prediabetes after a single-dose and after a month of daily raspberry consumption. Their targeted analyses revealed that several metabolite groups including ACN, Uros, ph-γ-VLs and phenolic acids were significantly changed after raspberry consumption. Cya-3-sophoroside (cya-3-sop), cya-3-rut, and cya-3-glu were found as the major ACN. Despite ACN being established as the most abundant (poly)phenols in berries, they inherently suffer from low bioavailability. They are also structurally unstable and readily degrade into smaller phenolic metabolites, making them problematic as BFIs. There are also no specific ACN for each individual berry, and ACN are largely common across red and purple foods such as red grapes, red wine, red apples, red onions, aubergines, or plums. One study^79^ performed targeted metabolomics in participants with obesity after 8 weeks of frozen raspberry consumption. They reported changes in 10 plasma metabolites (4 endogenous and 6 exogenous). The exogenous metabolites were made up of triacylglycerols, glycerophospholipids, and cholesterol esters (Table 1). These compounds are omnipresent and can be found across a wide range of animal and plant-based products rendering them ineffective BFIs. Lastly, Roberts et al.^80^ focused on metabolism of ellagitannins in male individuals upon 4 w consumption of raspberry products and quantified Uro A-D and dimethylellagic acid (DMEA) in plasma and urine. They reported an increase in all urolithins in urine, but only Uro A and C increased in plasma. In addition to Uro A, the author supports the consideration of DMEA as a good biomarker for ellagitannins exposure. Although DMEA may be specific to ellagitannins, it is still difficult to propose DMEA as the sole BFI for raspberry intake due to the ubiquitous nature of ellagitannins across many Rubus species, strawberry, walnuts, and many other foods widely abundant in the diet. As for Uros, they have been previously proposed as strong candidates as part of a multimetabolite panel for raspberry due to their specificity. These Uros include Uro A, Uro A glucuronide, Isouro A, Isouro A glucuronide, Uro B, Uro B glucuronide, and Uro C. However, Uro A and its derivatives are more likely to be good biomarkers as they are produced by the majority of the population, while Isouro A and uro B are only produced by some people.^81^ The combination of Uros with raspberry ketone metabolites as part of a multimarker panel may be specific enough to distinguish raspberry consumption from other foods such as strawberries. More efforts are needed to investigate the presence and metabolic fate of raspberry ketones in humans and to determine whether they may be suitable biomarkers for raspberry intake.

#### 3.2.4 Strawberries

Twenty papers were included investigating biomarkers of strawberry intake in the review of Ulaszewska et al.^19^ Pelargonidin and Uros derivatives were suggested as BFIs of strawberry. Besides, aromatic compounds such as furaneol and mesifurane were also proposed to be used as BFIs for strawberries. However, all of these metabolites lack specificity. A multi-BFIs including pelargonidin glucuronides, Uros, furaneol, or mesifurane derivatives was proposed as an adequate biomarker for strawberry intake. One drawback of this proposed approach is the lack of commercially available standards for pelargonidin glucuronide. In this review, 3 additional studies investigating biomarkers of strawberry intake were found.^82−84^ All were chronic interventions (≥4 w) that used reconstituted freeze-dried strawberry powder (25–26 g) as treatment. In Zhao et al.,^84^ a total of 81 bile acids were analyzed in plasma, urine and feces collectively after 4 w of strawberry consumption. Several secondary bile acids, deoxycholic acid, lithocholic acid (and their glycine conjugates), and glycoursodeoxycholic acid decreased upon intervention. While interesting, it is difficult to recommend primary bile acids as BFIs due to them being internally synthesized.^85^ Even though secondary bile acids are regarded as bacterial metabolites of the gut microbiota,^86^ they still lack specificity due to their endogenous nature. After 4 weeks of intake, Huang et al. identified 17 plasma phenolic metabolites in patients with hypercholesterolemia.^82^ These included various phenolic acid groups and their derivatives (Table 1) which have already been ruled out as candidates in the previous review for being common metabolites for many fruits and vegetables. In the last study,^83^ pelargonidin-3-glucuronide (pel-3-gluc) found in both plasma and urine further reinforces its standing as a strong BFI candidate for strawberries as proposed in the previous review alongside Uro A detected in urine samples. In summary, no additional strawberry BFI candidates were found to fit the proposed multimetabolite panel. Generally, the copresence of Uros and ACN derivatives in plasma and urine could be considered as an indicator of strawberry and/or raspberry intake. However, the timing of sample collection should be taken into consideration, as Uros and ACN have very different half-lives in blood and urine. As mentioned previously, ACN can rapidly degrade and disappear from the circulation within 6h, while Uros have a *C*_max_ of 24h post consumption. Furthermore, the high interindividual variability in the metabolism of Uros needs to be taken into consideration, in particular for Uro B and Isouro A derivatives, as it is estimated that in adulthood, only between 15% to 45% of the population produce such metabolites after ellagitannin consumption.^81^ This is an important limitation when they are used as biomarkers of berry consumption. As it currently stands, the panel consists of pelargonidin, pelargonidin glucuronide, mesifurane sulfate, furaneol glucuronide, and furaneol sulfate. These metabolites are reasonably unique to strawberries and together with Uro A derivatives to form the proposed multi-BFIs for strawberries (Table 2).

**Table 2:** Summary of Proposed Biomarkers of Berry Intake^a^

Berries	Suggested BFIs
Blueberries	No specific BFIs
	Suggested multibiomarker panel:
	Hippuric acid
	Malvidin glycosides
Cranberries	No specific BFIs
	Suggested multibiomarker panel:
	Peonidin and cyanidin glycosides
	ph-γ-VL- sulfate and glucuronide conjugates
Raspberries	No specific BFIs
	Suggested multibiomarker panel:
	Raspberry ketone sulfate/glucuronide
	Uro A/Uro A gluc
Strawberries	No specific BFIs
	Suggested multibiomarker panel:
	Pel/Pel-3-gluc
	Uro A/Uro A-gluc
	Furaneol glucuronide/Furaneol sulfate/Mesifurane sulfate
Blackcurrants	No specific BFIs or multibiomarker panel available
Blackberries	No specific BFIs or multibiomarker panel available

a ph-γ-VL: phenyl-*γ-*valerolactone, Uro A: urolithin A, Uro A gluc: urolithin A glucuronide, Pel: pelargonidin, Pel-3-gluc: pelargonidin-3-glucuronide.

#### 3.2.5 Blackcurrants

Eight studies^17,87−94^ investigating blackcurrant biomarkers of intake were discussed in the previous review. Two main ACN (cya-3-rut and delp-3-rut) were identified in urine. In plasma, these two ACN were also identified alongside cya-3-glu and del-3-glu. There were no bioavailability studies conducted on other blackcurrant phytochemicals in humans, which warrants the need for future clinical trials on blackcurrants and BFIs. In our review, only one additional study explored the bioavailability of plasma phenolic acids after an acute single-dose intake of blackcurrant.^95^ In this study, gallic acid (GA) and protocatechuic acid (PCA) increased in plasma after blackcurrant consumption. However, the participants in this trial were not asked to follow a restricted low (poly)phenol diet during the trial, and GA and PCA were also detected in their baseline plasma. GA and PCA are phenolics compounds that appears in biofluid samples as a result of gut microbiota metabolism after the consumption of many other (poly)phenol-rich fruits and foods.^96,97^ In line with the previous review, specific BFIs for blackcurrant intake are still lacking. Further studies are much encouraged to identify new candidates for blackcurrant intake.

#### 3.2.6 Blackberries

Five studies^98−102^ were included in the previous review to examine BFIs of blackberry intake. Like other Rubus fruits, blackberries are rich in ellagitannins and ACN. The main metabolites detected in plasma and urine in these studies were derivatives of cyanidins and ellagitannins, such as cya-3-gluc, cya-3-glucoside, cya-3-rut, Uro A gluc, Uro B gluc, and ellagic acid. Nevertheless, these metabolites are not specific to blackberries, as previously discussed for raspberries and strawberries. The authors of the previous systematic review suggested evaluating the Rubus fruits as a group and considering ellagitannins that conjugated with the ester of sanguisorboyl group as BFI. In this systematic review, only one small pilot study was found which evaluated the metabolic fate of blackberry (poly)phenols on 2 participants who consumed a blackberry juice as single dose.^103^ They detected sulfated cyanidin derivatives in 24 h urine that are not specific to blackberry. Thus, we still need more studies, ideally with untargeted metabolomics, to discover the potential BFIs of blackberries.

### 3.3 Final Remarks and Implications

**Figure 2:**
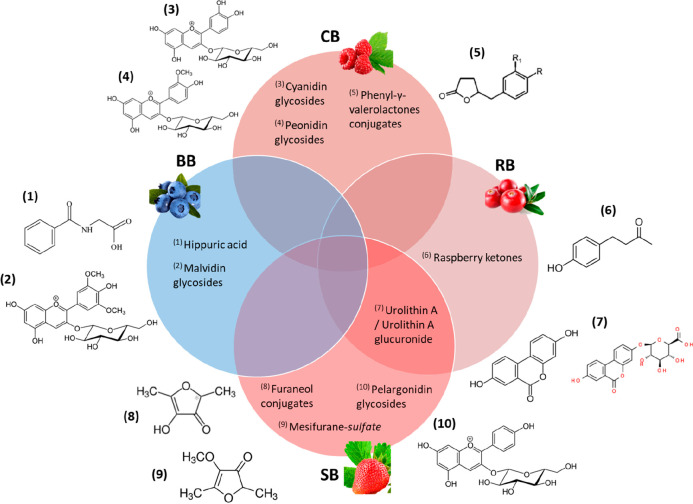
Venn diagram illustrating previously proposed BFIs alongside the new BFIs suggested in the current systematic review.

This systematic review provides an update on the current knowledge on biomarkers of berry intake. The previous review established tentative multi-BFI panels for cranberries, raspberries, and strawberries but failed to find suitable metabolite candidates for blackcurrants, blueberries, and blackberries. The results of our search are generally aligned with their findings. In addition, we propose a potential multi-BFI panel for blueberries consisting of hippuric acid and malvidin glycosides. We however acknowledge the limitation of the use of intact anthocyanins as biomarkers of intake due to their low bioavailability in biofluids and their natural instability. Our search confirms that BFIs specific to blackcurrants and blackberries have yet to be discovered. Figure 2 depicts a summary of our newly proposed BFIs on top of previous suggestions for intake of blueberries, cranberries, raspberries, and strawberries.

Berries can be consumed in different forms such as fresh, freeze-dried, juiced, and frozen. Freeze-drying is a technique that has shown to preserve (poly)phenols content without significant biotransformation.^104^ Although administering berries in different formats may have a significant impact on bioavailability due to the effects of the food structure and the food matrix,^105^ this is unlikely to have meaningful effects on the metabolism of berry (poly)phenols, although this needs to be confirmed in future studies. Another important aspect to consider when investigating biomarkers of intake in acute and chronic consumption studies is that changes in the gut microbiota population or diversity due to continuous exposure to berry (poly)phenols may lead to changes in gut microbial metabolism and therefore changes in the metabolites found in biofluids. Therefore, both acute assessments, which have the advantage of a much better control over background diet, coupled with sustained or long-term monitoring is recommended.

In the current systematic review, 11 studies were conducted in healthy individuals while 7 were conducted in individuals at risk of chronic diseases or under medication. Currently, little is known about how medication or pathological states may affect the metabolome upon consumption of berries and whether this differs from healthy individuals. To maximize the general applicability of biomarkers of intake, it is crucial to validate them in diverse study populations. Across the studies reviewed, we also noticed a huge variation in the sampling interval used to collect biological samples. When optimizing detection of (poly)phenols metabolites, it necessary to consider their individual time to reach maximum concentration (*T*_max_) and half-life (*T*_1/2_). These are crucial points to help inform the optimum sampling interval for maximizing detection and quantification. It can become challenging or problematic when many metabolites are simultaneously being monitored especially in a multi-BFI approach in which the sampling interval for each metabolite has to be optimized.

BFIs detected in this systematic review included both blood and urine metabolites. The assessment of (poly)phenols metabolites excreted through urine is commonly used and typically consistent with the data obtained from plasma.^106^ However, 24-h urine sampling offers advantages over plasma measurements, providing a more accurate evaluation of total (poly)phenols absorption and better tracking of (poly)phenols with short and long half-lives.^106^ On the other hand, a study suggested that spot urine not only provides a lower collection burden for participants, but it is considered a viable alternative for 24 h urine for dietary exposure biomarkers.^107^ While urine sampling does not capture (poly)phenols excreted through alternative routes, combining plasma sampling with urine sampling may be the preferable approach for measuring (poly)phenols as biomarkers of intake.

Many of the detected metabolites in the studies reviewed here were present in a conjugated form. The metabolism of (poly)phenols involves the conversion of (poly)phenols into simple aglycones in the gastrointestinal tract, followed by phase II metabolism with additional modifications such as methylation, sulfation, and glucuronidation. Enzymes like Catechol-O-methyltransferase (COMT), sulfotransferases (SULT), and uridine-5′-diphosphate glucuronosyltransferases (UGT) play essential roles in these processes, transferring methyl, sulfate, and glucuronic acid groups to the aglycones, respectively.^108,109^ The specific conjugates formed can vary based on the compound’s nature and available sites for conjugation.^110^

In conclusion, there is currently no singular biomarker that can be confidently linked to individual berries. The multibiomarker approach appears to be the best option for estimating berry consumption, although concerns about the specificity of current proposed panels still exist for all berries investigated here. Untargeted metabolomic studies coupled to well conducted acute and chronic feeding studies using adequate biofluid sample collection in different study populations may help improve current knowledge in berry biomarker discovery.

## Supporting Information Available

Systematic search was conducted using three electronic databases, in accordance with the PRISMA statement, to identify relevant studies: details of the search strategy and the databases searched (PDF)

## References

[ref1] Mithril C.; Dragsted L. O.; Meyer C.; Tetens I.; Biltoft-Jensen A.; Astrup A. Dietary composition and nutrient content of the New Nordic Diet. Public Health Nutr. 2013, 16 (5), 777–785. 10.1017/S1368980012004521. 23089239 PMC10271429

[ref2] Battino M.; Forbes-Hernández T. Y.; Gasparrini M.; Afrin S.; Cianciosi D.; Zhang J.; Manna P. P.; Reboredo-Rodríguez P.; Giampieri F.; et al. Relevance of functional foods in the Mediterranean diet: the role of olive oil,berries and honey in the prevention of cancer and cardiovascular diseases. Crit Rev. Food Sci. Nutr. 2019, 59 (6), 893–920. 10.1080/10408398.2018.1526165. 30421983

[ref3] Li D.; Wang P.; Luo Y.; Zhao M.; Chen F. Health benefits of anthocyanins and molecular mechanisms: Update from recent decade. Crit Rev. Food Sci. Nutr. 2017, 57 (8), 1729–1741. 10.1080/10408398.2015.1030064. 26192537

[ref4] Golovinskaia O.; Wang C. K. Review of functional and pharmacological activities of berries. Molecules 2021, 26 (13), 3904 10.3390/molecules26133904. 34202412 PMC8271923

[ref5] Behrendt I.; Röder I.; Will F.; Mostafa H.; Gonzalez-Dominguez R.; Meroño T.; Andres-Lacueva C.; Fasshauer M.; Kuntz S. Influence of Plasma-Isolated Anthocyanins and Their Metabolites on Cancer Cell Migration (HT-29 and Caco-2) In Vitro: Results of the ATTACH Study. Antioxidants 2022, 11 (7), 1341 10.3390/antiox11071341. 35883834 PMC9311669

[ref6] Amin A. M. Metabolomics Applications in Coronary Artery Disease Personalized Medicine, 1st ed.; Elsevier Inc., 2021; Vol 102. 10.1016/bs.acc.2020.08.003. 34044911

[ref7] Ulaszewska M. M.; Weinert C. H.; Trimigno A.; Portmann R.; Andres Lacueva C.; Badertscher R.; Brennan L.; Brunius C.; Vergères G.; et al. Nutrimetabolomics: An Integrative Action for Metabolomic Analyses in Human Nutritional Studies. Mol. Nutr Food Res. 2019, 63 (1), 1–38. 10.1002/mnfr.201800384. 30176196

[ref8] Rangel-Huerta O. D. Nutrimetabolomics: A step further into personalized nutrition. Nor Tidsskr ernæring 2018, 16 (4), 1–10. 10.18261/ntfe.16.4.11.

[ref9] Di Pede G.; Mena P.; Bresciani L.; Achour M.; Lamuela-Raventós R. M.; Estruch R.; Landberg R.; Kulling S. E.; Del Rio D. Revisiting the bioavailability of flavan-3-ols in humans: A systematic review and comprehensive data analysis. Mol. Aspects Med. 2022, (July), 101146 10.1016/j.mam.2022.101146. 36207170

[ref10] Favari C.; Mena P.; Curti C.; Istas G.; Heiss C.; Del Rio D.; Rodriguez-Mateos A. Kinetic profile and urinary excretion of phenyl-γ-valerolactones upon consumption of cranberry: A dose-response relationship. Food Funct. 2020, 11 (5), 3975–3985. 10.1039/D0FO00806K. 32396592

[ref11] Mena P.; Favari C.; Acharjee A.; Chernbumroong S.; Bresciani L.; Curti C.; Brighenti F.; Heiss C.; Del Rio D.; et al. Metabotypes of flavan-3-ol colonic metabolites after cranberry intake: elucidation and statistical approaches. Eur. J. Nutr. 2022, 61 (3), 1299–1317. 10.1007/s00394-021-02692-z. 34750642 PMC8921115

[ref12] Istas G.; Feliciano R. P.; Weber T.; Garcia-villalba R.; Heiss C.; Rodriguez-mateos A.; et al. Plasma urolithin metabolites correlate with improvements in endothelial function after red raspberry consumption: A double-blind randomized controlled trial. Arch. Biochem. Biophys. 2018, 651, 43 10.1016/j.abb.2018.05.016. 29802820

[ref13] Truchado P.; Larrosa M.; García-Conesa M. T.; Cerdá B.; Vidal-Guevara M. L.; Tomás-Barberán F. A.; Espín J. C. Strawberry processing does not affect the production and urinary excretion of urolithins, ellagic acid metabolites, in humans. J. Agric. Food Chem. 2012, 60 (23), 5749–5754. 10.1021/jf203641r. 22126674

[ref14] Cerdá B.; Tomás-Barberán F. A.; Espín J. C. Metabolism of antioxidant and chemopreventive ellagitannins from strawberries, raspberries, walnuts, and oak-aged wine in humans: Identification of biomarkers and individual variability. J. Agric. Food Chem. 2005, 53 (2), 227–235. 10.1021/jf049144d. 15656654

[ref15] Cuparencu C. S.; Andersen M. B. S.; Gürdeniz G.; Schou S. S.; Mortensen M. W.; Raben A.; Astrup A.; Dragsted L. O. Identification of urinary biomarkers after consumption of sea buckthorn and strawberry, by untargeted LC–MS metabolomics: a meal study in adult men. Metabolomics. 2016, 12 (2), 1–20. 10.1007/s11306-015-0934-0.

[ref16] Mazza G.; Kay C. D.; Cottrell T.; Holub B. J. Absorption of anthocyanins from blueberries and serum antioxidant status in human subjects. J. Agric. Food Chem. 2002, 50 (26), 7731–7737. 10.1021/jf020690l. 12475297

[ref17] McGhie T. K.; Ainge G. D.; Barnett L. E.; Cooney J. M.; Jensen D. J. Anthocyanin glycosides from berry fruit are absorbed and excreted unmetabolized by both humans and rats. J. Agric. Food Chem. 2003, 51 (16), 4539–4548. 10.1021/jf026206w. 14705874

[ref18] McNamara A. E.; Walton J.; Flynn A.; Nugent A. P.; McNulty B. A.; Brennan L. The Potential of Multi-Biomarker Panels in Nutrition Research: Total Fruit Intake as an Example. Front Nutr. 2021, 7 (January), 1–8. 10.3389/fnut.2020.577720. PMC7840580 33521031

[ref19] Ulaszewska M.; Garcia-Aloy M.; Vázquez-Manjarrez N.; Soria-Florido M. T.; Llorach R.; Mattivi F.; Manach C. Food intake biomarkers for berries and grapes. Genes Nutr. 2020, 15 (1), 17 10.1186/s12263-020-00675-z. 32967625 PMC7509942

[ref20] Rodriguez-Mateos A.; Istas G.; Boschek L.; Feliciano R. P.; Mills C. E.; Boby C.; Gomez-Alonso S.; Milenkovic D.; Heiss C. Circulating Anthocyanin Metabolites Mediate Vascular Benefits of Blueberries: Insights from Randomized Controlled Trials, Metabolomics, and Nutrigenomics. Journals Gerontol - Ser. A Biol. Sci. Med. Sci. 2019, 74 (7), 967–976. 10.1093/gerona/glz047. 30772905

[ref21] Kalt W.; McDonald J. E.; Vinqvist-Tymchuk M. R.; Liu Y.; Fillmore S. A. E. Human anthocyanin bioavailability: effect of intake duration and dosing. Food Funct. 2017, 8 (12), 4563–4569. 10.1039/C7FO01074E. 29115354

[ref22] Sobolev A. P.; Ciampa A.; Ingallina C.; Mannina L.; Capitani D.; Ernesti I.; Maggi E.; Businaro R.; Pinto A. Blueberry-based meals for obese patients with metabolic syndrome: A multidisciplinary metabolomic pilot study. Metabolites. 2019, 9 (7), 1–17. 10.3390/metabo9070138. PMC6680695 31295937

[ref23] Bharat D.; Cavalcanti R. R. M.; Petersen C.; Begaye N.; Cutler B. R.; Costa M. M. A.; Ramos R.; Ferreira M. R.; Anandh Babu P. V. Blueberry Metabolites Attenuate Lipotoxicity-Induced Endothelial Dysfunction. Mol. Nutr Food Res. 2018, 62 (2), 1–8. 10.1002/mnfr.201700601. PMC8162272 29024402

[ref24] de Mello V. D. F.; Lankinen M. A.; Lindström J.; Puupponen-Pimiä R.; Laaksonen D. E.; Pihlajamäki J.; Lehtonen M.; Uusitupa M.; Hanhineva K.; et al. Fasting serum hippuric acid is elevated after bilberry (Vaccinium myrtillus) consumption and associates with improvement of fasting glucose levels and insulin secretion in persons at high risk of developing type 2 diabetes. Mol. Nutr Food Res. 2017, 61 (9), 1–8. 10.1002/mnfr.201700019. 28556578

[ref25] Tang J. S.; Vissers M. C. M.; Anderson R. F.; Sreebhavan S.; Bozonet S. M.; Scheepens A.; Melton L. D. Bioavailable Blueberry-Derived Phenolic Acids at Physiological Concentrations Enhance Nrf2-Regulated Antioxidant Responses in Human Vascular Endothelial Cells. Mol. Nutr Food Res. 2018, 62 (5), 1–12. 10.1002/mnfr.201700647. 29278300

[ref26] Zhong S.; Sandhu A.; Edirisinghe I.; Burton-Freeman B. Characterization of Wild Blueberry Polyphenols Bioavailability and Kinetic Profile in Plasma over 24-h Period in Human Subjects. Mol. Nutr Food Res. 2017, 61 (12), 1–13. 10.1002/mnfr.201700405. 28887907

[ref27] Feliciano R. P.; Istas G.; Heiss C.; Rodriguez-Mateos A. Plasma and urinary phenolic profiles after acute and repetitive intake of wild blueberry. Molecules. 2016, 21 (9), 14–16. 10.3390/molecules21091120. PMC6273248 27571052

[ref28] Bensalem J.; Dudonné S.; Etchamendy N.; Pellay H.; Amadieu C.; Gaudout D.; Dubreuil S.; Paradis M. E.; Pallet V.; et al. Polyphenols from Grape and Blueberry Improve Episodic Memory in Healthy Elderly with Lower Level of Memory Performance: A Bicentric Double-Blind, Randomized, Placebo-Controlled Clinical Study. Journals Gerontol - Ser. A Biol. Sci. Med. Sci. 2019, 74 (7), 996–1007. 10.1093/gerona/gly166. 30032176

[ref29] Langer S.; Kennel A.; Lodge J. K. The influence of juicing on the appearance of blueberry metabolites 2 h after consumption: A metabolite profiling approach. Br. J. Nutr. 2018, 119 (11), 1233–1244. 10.1017/S0007114518000855. 29770756

[ref30] McNamara R. K.; Kalt W.; Shidler M. D.; McDonald J.; Summer S. S.; Stein A. L.; Stover A. N.; Krikorian R. Cognitive response to fish oil, blueberry, and combined supplementation in older adults with subjective cognitive impairment. Neurobiol Aging. 2018, 64, 147–156. 10.1016/j.neurobiolaging.2017.12.003. 29458842 PMC5822748

[ref31] Ancillotti C.; Ulaszewska M.; Mattivi F.; Del Bubba M. Untargeted Metabolomics Analytical Strategy Based on Liquid Chromatography/Electrospray Ionization Linear Ion Trap Quadrupole/Orbitrap Mass Spectrometry for Discovering New Polyphenol Metabolites in Human Biofluids after Acute Ingestion of Vaccinium myrti. J. Am. Soc. Mass Spectrom. 2019, 30 (3), 381–402. 10.1007/s13361-018-2111-y. 30506347

[ref32] Mueller D.; Jung K.; Winter M.; Rogoll D.; Melcher R.; Richling E. Human intervention study to investigate the intestinal accessibility and bioavailability of anthocyanins from bilberries. Food Chem. 2017, 231, 275–286. 10.1016/j.foodchem.2017.03.130. 28450007

[ref33] Wu X.; Cao G.; Prior R. L. Absorption and metabolism of anthocyanins in elderly women after consumption of elderberry or blueberry. J. Nutr. 2002, 132 (7), 1865–1871. 10.1093/jn/132.7.1865. 12097661

[ref34] Arevström L.; Bergh C.; Landberg R.; Wu H.; Rodriguez-Mateos A.; Waldenborg M.; Magnuson A.; Blanc S.; Fröbert O. Freeze-dried bilberry (Vaccinium myrtillus) dietary supplement improves walking distance and lipids after myocardial infarction: an open-label randomized clinical trial. Nutr. Res. (N.Y.) 2019, 62, 13–22. 10.1016/j.nutres.2018.11.008. 30803503

[ref35] Kalt W.; McDonald J. E.; Liu Y.; Fillmore S. A. E. Flavonoid Metabolites in Human Urine during Blueberry Anthocyanin Intake. J. Agric. Food Chem. 2017, 65 (8), 1582–1591. 10.1021/acs.jafc.6b05455. 28150498

[ref36] Kalt W.; Liu Y.; McDonald J. E.; Vinqvist-Tymchuk M. R.; Fillmore S. A. E. Anthocyanin metabolites are abundant and persistent in human urine. J. Agric. Food Chem. 2014, 62 (18), 3926–3934. 10.1021/jf500107j. 24432743

[ref37] Del Bo C.; Riso P.; Brambilla A.; Gardana C.; Rizzolo A.; Simonetti P.; Bertolo G.; Klimis-Zacas D.; Porrini M. Blanching Improves Anthocyanin Absorption from Highbush. J. Agric. Food Chem. 2012, 60, 9298–9304. 10.1021/jf3021333. 22906096

[ref38] Curtis P. J; van der Velpen V.; Berends L.; Jennings A.; Feelisch M.; Umpleby A M.; Evans M.; Fernandez B. O; Meiss M. S; Minnion M.; Potter J.; Minihane A.-M.; Kay C. D; Rimm E. B; Cassidy A. Blueberries improve biomarkers of cardiometabolic function in participants with metabolic syndrome — results from a 6-month, double-blind, randomized controlled trial. Am. J. Clin. Nutr. 2019, 110 (5), 1535–1545. 10.1093/ajcn/nqy380. PMC6537945 31136659

[ref39] Rousseau M.; Horne J.; Guénard F.; de Toro-Martín J.; Garneau V.; Guay V.; Kearney M.; Pilon G.; Vohl M. C. An 8-week freeze-dried blueberry supplement impacts immune-related pathways: a randomized, double-blind placebo-controlled trial. Genes Nutr. 2021, 16 (1), 7 10.1186/s12263-021-00688-2. 34000994 PMC8130140

[ref40] Curtis P. J.; Berends L.; van der Velpen V.; Jennings A.; Haag L.; Chandra P.; Kay C. D.; Rimm E. B.; Cassidy A. Blueberry anthocyanin intake attenuates the postprandial cardiometabolic effect of an energy-dense food challenge: Results from a double blind, randomized controlled trial in metabolic syndrome participants. Clin Nutr. 2022, 41 (1), 165–176. 10.1016/j.clnu.2021.11.030. 34883305 PMC8757535

[ref41] Rutledge G. A.; Sandhu A. K.; Miller M. G.; Edirisinghe I.; Burton-Freeman B. B.; Shukitt-Hale B. Blueberry phenolics are associated with cognitive enhancement in supplemented healthy older adults. Food Funct. 2021, 12 (1), 107–118. 10.1039/D0FO02125C. 33331835

[ref42] Renai L.; Ancillotti C.; Ulaszewska M.; Garcia-Aloy M.; Mattivi F.; Bartoletti R.; Del Bubba M. Comparison of chemometric strategies for potential exposure marker discovery and false-positive reduction in untargeted metabolomics: application to the serum analysis by LC-HRMS after intake of Vaccinium fruit supplements. Anal Bioanal Chem. 2022, 414 (5), 1841–1855. 10.1007/s00216-021-03815-5. 35028688

[ref43] Barfoot K. L.; Istas G.; Feliciano R. P.; Lamport D. J.; Riddell P.; Rodriguez-Mateos A.; Williams C. M. Effects of daily consumption of wild blueberry on cognition and urinary metabolites in school-aged children: a pilot study. Eur. J. Nutr. 2021, 60 (8), 4263–4278. 10.1007/s00394-021-02588-y. 34023938 PMC8572198

[ref44] Mostafa H.; Amin A. M.; Teh C. H.; Murugaiyah V.; Arif N. H.; Ibrahim B. Metabolic phenotyping of urine for discriminating alcohol-dependent from social drinkers and alcohol-naive subjects. Drug Alcohol Depend. 2016, 169, 80–84. 10.1016/j.drugalcdep.2016.10.016. 27788404

[ref45] Herraiz T.; Galisteo J. Tetrahydro-β-carboline alkaloids that occur in foods and biological systems act as radical scavengers and antioxidants in the ABTS assay. Free Radic Res. 2002, 36 (8), 923–928. 10.1080/1071576021000005762. 12420751

[ref46] Lotito S. B.; Frei B. Consumption of flavonoid-rich foods and increased plasma antioxidant capacity in humans: Cause, consequence, or epiphenomenon?. Free Radic Biol. Med. 2006, 41 (12), 1727–1746. 10.1016/j.freeradbiomed.2006.04.033. 17157175

[ref47] Medjakovic S.; Jungbauer A. Pomegranate: A fruit that ameliorates metabolic syndrome. Food Funct. 2013, 4 (1), 19–39. 10.1039/C2FO30034F. 23060097

[ref48] Penczynski K. J.; Krupp D.; Bring A.; Bolzenius K.; Remer T.; Buyken A. E. Relative validation of 24-h urinary hippuric acid excretion as a biomarker for dietary flavonoid intake from fruit and vegetables in healthy adolescents. Eur. J. Nutr. 2017, 56 (2), 757–766. 10.1007/s00394-015-1121-9. 26658765

[ref49] Yuan L.; Muli S.; Huybrechts I.; Nöthlings U.; Ahrens W.; Scalbert A.; Floegel A. Assessment of Fruit and Vegetables Intake with Biomarkers in Children and Adolescents and Their Level of Validation: A Systematic Review. Metabolites. 2022, 12 (2), 1–17. 10.3390/metabo12020126. PMC8876138 35208201

[ref50] Di Lorenzo C.; Colombo F.; Biella S.; Stockley C.; Restani P. Polyphenols and human health: The role of bioavailability. Nutrients. 2021, 13 (1), 1–30. 10.3390/nu13010273. PMC7833401 33477894

[ref51] Peron G.; Sut S.; Pellizzaro A.; Brun P.; Voinovich D.; Castagliuolo I.; Dall’Acqua S. The antiadhesive activity of cranberry phytocomplex studied by metabolomics: Intestinal PAC-A metabolites but not intact PAC-A are identified as markers in active urines against uropathogenic Escherichia coli. Fitoterapia. 2017, 122 (June), 67–75. 10.1016/j.fitote.2017.08.014. 28844930

[ref52] Feliciano R. P.; Boeres A.; Massacessi L.; Istas G.; Ventura M. R.; Nunes Dos Santos C.; Heiss C.; Rodriguez-Mateos A. Identification and quantification of novel cranberry-derived plasma and urinary (poly)phenols. Arch. Biochem. Biophys. 2016, 599, 31–41. 10.1016/j.abb.2016.01.014. 26836705

[ref53] Feliciano R. P.; Mills C. E.; Istas G.; Heiss C.; Rodriguez-Mateos A. Absorption, metabolism and excretion of cranberry (poly)phenols in humans: A dose response study and assessment of inter-individual variability. Nutrients 2017, 9 (3), 268 10.3390/nu9030268. 28287476 PMC5372931

[ref54] Wang Y.; Singh A. P.; Nelson H. N.; Kaiser A. J.; Reker N. C.; Hooks T. L.; Wilson T.; Vorsa N. Urinary Clearance of Cranberry Flavonol Glycosides in Humans. J. Agric. Food Chem. 2016, 64 (42), 7931–7939. 10.1021/acs.jafc.6b03611. 27690414

[ref55] Ohnishi R.; Ito H.; Kasajima N.; Kaneda M.; Kariyama R.; Kumon H.; Hatano T.; Yoshida T. Urinary excretion of anthocyanins in humans after cranberry juice ingestion. Biosci Biotechnol Biochem. 2006, 70 (7), 1681–1687. 10.1271/bbb.60023. 16861803

[ref56] Baron G.; Altomare A.; Regazzoni L.; Fumagalli L.; Artasensi A.; Borghi E.; Ottaviano E.; Del Bo C.; Aldini G.; et al. Profiling Vaccinium macrocarpon components and metabolites in human urine and the urine ex-vivo effect on Candida albicans adhesion and biofilm-formation. Biochem. Pharmacol. 2020, 173, 113726 10.1016/j.bcp.2019.113726. 31778647

[ref57] Wang C.; Zuo Y.; Vinson J. A.; Deng Y. Absorption and excretion of cranberry-derived phenolics in humans. Food Chem. 2012, 132 (3), 1420–1428. 10.1016/j.foodchem.2011.11.131. 29243631

[ref58] Liu H.; Garrett T. J.; Su Z.; Khoo C.; Gu L. UHPLC-Q-Orbitrap-HRMS-based global metabolomics reveal metabolome modifications in plasma of young women after cranberry juice consumption. J. Nutr Biochem. 2017, 45, 67–76. 10.1016/j.jnutbio.2017.03.007. 28433923

[ref59] Milbury P. E.; Vita J. A.; Blumberg J. B. Anthocyanins are bioavailable in humans following an acute dose of cranberry juice. J. Nutr. 2010, 140 (6), 1099–1104. 10.3945/jn.109.117168. 20375263

[ref60] Chew B.; Mathison B.; Kimble L.; McKay D.; Kaspar K.; Khoo C.; Chen C. Y. O.; Blumberg J. Chronic consumption of a low calorie, high polyphenol cranberry beverage attenuates inflammation and improves glucoregulation and HDL cholesterol in healthy overweight humans: a randomized controlled trial. Eur. J. Nutr. 2019, 58 (3), 1223–1235. 10.1007/s00394-018-1643-z. 29476238 PMC6499871

[ref61] Zhang K.; Zuo Y. GC-MS Determination of Flavonoids and Phenolic and Benzoic Acids in Human Plasma after Consumption of Cranberry Juice. J. Agric. Food Chem. 2004, 52 (2), 222–227. 10.1021/jf035073r. 14733499

[ref62] Walsh J. M.; Ren X.; Zampariello C.; Polasky D. A.; McKay D. L.; Blumberg J. B. Chen CYO. Liquid chromatography with tandem mass spectrometry quantification of urinary proanthocyanin A2 dimer and its potential use as a biomarker of cranberry intake. J. Sep Sci. 2016, 39, 342–349. 10.1002/jssc.201500922. 26573891

[ref63] Rodriguez-Mateos A.; Feliciano R. P.; Boeres A.; Weber T.; dos Santos C. N.; Ventura M. R.; Heiss C. Cranberry (poly)phenol metabolites correlate with improvements in vascular function: A double-blind, randomized, controlled, dose-response, crossover study. Mol. Nutr Food Res. 2016, 60 (10), 2130–2140. 10.1002/mnfr.201600250. 27242317

[ref64] Heiss C.; Istas G.; Feliciano R. P.; Weber T.; Wang B.; Favari C.; Mena P.; Del Rio D.; Rodriguez-Mateos A. Daily consumption of cranberry improves endothelial function in healthy adults: a double blind randomized controlled trial. Food Funct. 2022, 13 (7), 3812–3824. 10.1039/D2FO00080F. 35322843

[ref65] Liu H.; Garrett T. J.; Su Z.; Khoo C.; Zhao S.; Gu L. Modifications of the urinary metabolome in young women after cranberry juice consumption were revealed using the UHPLC-Q-orbitrap-HRMS-based metabolomics approach. Food Funct. 2020, 11 (3), 2466–2476. 10.1039/C9FO02266J. 32133462

[ref66] Zhao S.; Liu H.; Su Z.; Khoo C.; Gu L. Identifying Cranberry Juice Consumers with Predictive OPLS-DA Models of Plasma Metabolome and Validation of Cranberry Juice Intake Biomarkers in a Double-Blinded, Randomized, Placebo-Controlled, Cross-Over Study. Mol. Nutr Food Res. 2020, 64 (11), 1–15. 10.1002/mnfr.201901242. 32281738

[ref67] González-Domínguez R.; Urpi-Sarda M.; Jáuregui O.; Needs P. W.; Kroon P. A.; Andrés-Lacueva C. Quantitative Dietary Fingerprinting (QDF)-A Novel Tool for Comprehensive Dietary Assessment Based on Urinary Nutrimetabolomics. J. Agric. Food Chem. 2020, 68 (7), 1851 10.1021/acs.jafc.8b07023. 30799616

[ref68] González-Domínguez R.; Jáuregui O.; Queipo-Ortuño M. I.; Andrés-Lacueva C. Characterization of the Human Exposome by a Comprehensive and Quantitative Large-Scale Multianalyte Metabolomics Platform. Anal. Chem. 2020, 92 (20), 13767–13775. 10.1021/acs.analchem.0c02008. 32966057

[ref69] Ottaviani J. I.; Fong R.; Kimball J.; Ensunsa J. L.; Britten A.; Lucarelli D.; Luben R.; Grace P. B.; Kuhnle G. G. C.; et al. Evaluation at scale of microbiome-derived metabolites as biomarker of flavan-3-ol intake in epidemiological studies. Sci. Rep. 2018, 8 (1), 1–11. 10.1038/s41598-018-28333-w. 29959422 PMC6026136

[ref70] Ottaviani J. I.; Britten A.; Lucarelli D.; Luben R.; Mulligan A. A.; Lentjes M. A.; Fong R.; Gray N.; Kuhnle G. G. C. Biomarker-estimated flavan-3-ol intake is associated with lower blood pressure in cross-sectional analysis in EPIC Norfolk. Sci. Rep. 2020, 10 (1), 17964 10.1038/s41598-020-74863-7. 33087825 PMC7578063

[ref71] Kim Y. J.; Huh I.; Kim J. Y.; Park S.; Ryu S. H.; Kim K. B.; Kim S.; Park T.; Kwon O. Integration of traditional and metabolomics biomarkers identifies prognostic metabolites for predicting responsiveness to nutritional intervention against oxidative stress and inflammation. Nutrients. 2017, 9 (3), 233 10.3390/nu9030233. 28273855 PMC5372896

[ref72] Ludwig I. A.; Mena P.; Calani L.; Borges G.; Pereira-Caro G.; Bresciani L.; Del Rio D.; Lean M. E. J.; Crozier A. New insights into the bioavailability of red raspberry anthocyanins and ellagitannins. Free Radic Biol. Med. 2015, 89, 758–769. 10.1016/j.freeradbiomed.2015.10.400. 26475039

[ref73] Zhang X.; Sandhu A.; Edirisinghe I.; Burton-Freeman B. An exploratory study of red raspberry (: Rubus idaeus L.) (poly)phenols/metabolites in human biological samples. Food Funct 2018, 9 (2), 806–818. 10.1039/C7FO00893G. 29344587

[ref74] González-Barrio R.; Borges G.; Mullen W.; Crozier A. Bioavailability of anthocyanins and ellagitannins following consumption of raspberries by healthy humans and subjects with an ileostomy. J. Agric. Food Chem. 2010, 58 (7), 3933–3939. 10.1021/jf100315d. 20218618

[ref75] Sporstol S.; Scheline R. R. The metabolism of 4-(4-hydroxyphenyl)butan-2-one (raspberry ketone) in rats, Guinea-pigs and rabbits. Xenobiotica. 1982, 12 (4), 249–257. 10.3109/00498258209052463. 7113261

[ref76] Lloyd A. J.; Favé G.; Beckmann M.; Lin W.; Tailliart K.; Xie L.; Mathers J. C.; Draper J. Use of mass spectrometry fingerprinting to identify urinary metabolites after consumption of specific foods. Am. J. Clin. Nutr. 2011, 94 (4), 981–991. 10.3945/ajcn.111.017921. 21865330

[ref77] Zhang X.; Fan J.; Xiao D.; Edirisinghe I.; Burton-Freeman B. M.; Sandhu A. K. Pharmacokinetic Evaluation of Red Raspberry (Poly)phenols from Two Doses and Association with Metabolic Indices in Adults with Prediabetes and Insulin Resistance. J. Agric. Food Chem. 2021, 69 (32), 9238–9248. 10.1021/acs.jafc.1c02404. 34357772

[ref78] Zhang X.; Sandhu A.; Edirisinghe I.; Burton-Freeman B. M. Plasma and urinary (poly)phenolic profiles after 4-week red raspberry (rubus idaeus L.) intake with or without fructo-oligosaccharide supplementation. Molecules 2020, 25 (20), 4777 10.3390/molecules25204777. 33080934 PMC7594073

[ref79] Franck M.; de Toro-Martín J.; Garneau V.; Guay V.; Kearney M.; Pilon G.; Roy D.; Couture P.; Vohl M. C.; et al. Effects of daily raspberry consumption on immune-metabolic health in subjects at risk of metabolic syndrome: A randomized controlled trial. Nutrients. 2020, 12 (12), 1–20. 10.3390/nu12123858. PMC7767072 33348685

[ref80] Roberts K. M.; Grainger E. M.; Thomas-Ahner J. M.; Hinton A.; Gu J.; Riedl K.; Vodovotz Y.; Abaza R.; Clinton S. K.; et al. Dose-Dependent Increases in Ellagitannin Metabolites as Biomarkers of Intake in Humans Consuming Standardized Black Raspberry Food Products Designed for Clinical Trials. Mol. Nutr Food Res. 2020, 64 (10), 1–10. 10.1002/mnfr.201900800. PMC9496378 32112501

[ref81] García-Villalba R.; Giménez-Bastida J. A.; Cortés-Martín A.; Ávila-Gálvez MÁ; Tomás-Barberán F. A.; Selma M. V.; Espín J. C.; González-Sarrías A. Urolithins: a Comprehensive Update on their Metabolism, Bioactivity, and Associated Gut Microbiota. Mol. Nutr Food Res. 2022, 66 (21), 2101019 10.1002/mnfr.202101019. 35118817 PMC9787965

[ref82] Huang L.; Xiao D.; Zhang X.; Sandhu A. K.; Chandra P.; Kay C.; Edirisinghe I.; Burton-Freeman B. Strawberry Consumption, Cardiometabolic Risk Factors, and Vascular Function: A Randomized Controlled Trial in Adults with Moderate Hypercholesterolemia. J. Nutr. 2021, 151 (6), 1517–1526. 10.1093/jn/nxab034. 33758944

[ref83] Ezzat-Zadeh Z.; Henning S. M.; Yang J.; Woo S. L.; Lee R. P.; Huang J.; Thames G.; Gilbuena I.; Li Z.; et al. California strawberry consumption increased the abundance of gut microorganisms related to lean body weight, health and longevity in healthy subjects. Nutr. Res. (N.Y.) 2021, 85, 60–70. 10.1016/j.nutres.2020.12.006. 33450667

[ref84] Zhao A.; Zhang L.; Zhang X.; Edirisinghe I.; Burton-Freeman B. M.; Sandhu A. K. Comprehensive characterization of bile acids in human biological samples and effect of 4-week strawberry intake on bile acid composition in human plasma. Metabolites. 2021, 11 (2), 1–24. 10.3390/metabo11020099. PMC7916557 33578858

[ref85] Chiang J. Y. L.; Ferrell J. M. Bile acid metabolism in liver pathobiology. Gene Expr. 2018, 18 (2), 71–87. 10.3727/105221618X15156018385515. 29325602 PMC5954621

[ref86] Zeng H.; Umar S.; Rust B.; Lazarova D.; Bordonaro M. Secondary bile acids and short chain fatty acids in the colon: A focus on colonic microbiome, cell proliferation, inflammation, and cancer. Int. J. Mol. Sci. 2019, 20 (5), 1214 10.3390/ijms20051214. 30862015 PMC6429521

[ref87] Hollands W.; Brett G. M.; Radreau P.; Saha S.; Teucher B.; Bennett R. N.; Kroon P. A. Processing blackcurrants dramatically reduces the content and does not enhance the urinary yield of anthocyanins in human subjects. Food Chem. 2008, 108 (3), 869–878. 10.1016/j.foodchem.2007.11.052. 26065747

[ref88] Törrönen R.; McDougall G. J.; Dobson G.; Stewart D.; Hellström J.; Mattila P.; Pihlava J. M.; Koskela A.; Karjalainen R. Fortification of blackcurrant juice with crowberry: Impact on polyphenol composition, urinary phenolic metabolites, and postprandial glycemic response in healthy subjects. J. Funct Foods. 2012, 4 (4), 746–756. 10.1016/j.jff.2012.05.001.

[ref89] Jin Y.; Alimbetov D.; George T.; Gordon M. H.; Lovegrove J. A. A randomised trial to investigate the effects of acute consumption of a blackcurrant juice drink on markers of vascular reactivity and bioavailability of anthocyanins in human subjects. Eur. J. Clin Nutr. 2011, 65 (7), 849–856. 10.1038/ejcn.2011.55. 21540876

[ref90] Röhrig T.; Kirsch V.; Schipp D.; Galan J.; Richling E. Absorption of Anthocyanin Rutinosides after Consumption of a Blackcurrant (Ribes nigrum L). Extract. J. Agric Food Chem. 2019, 67 (24), 6792–6797. 10.1021/acs.jafc.9b01567. 31134806

[ref91] Erlund I.; Marniemi J.; Hakala P.; Alfthan G.; Meririnne E.; Aro A. Consumption of black currants, lingonberries and bilberries increases serum quercetin concentrations. Eur. J. Clin Nutr. 2003, 57 (1), 37–42. 10.1038/sj.ejcn.1601513. 12548295

[ref92] Nakamura Y.; Matsumoto H.; Morifuji M.; Iida H.; Takeuchi Y. Development and validation of a liquid chromatography tandem mass spectrometry method for simultaneous determination of four anthocyanins in human plasma after black currant anthocyanins ingestion. J. Agric. Food Chem. 2010, 58 (2), 1174–1179. 10.1021/jf9027365. 20028128

[ref93] Igwe E. O.; Charlton K. E.; Probst Y. C.; Kent K.; Netzel M. E. A systematic literature review of the effect of anthocyanins on gut microbiota populations. J. Hum Nutr Diet. 2019, 32 (1), 53–62. 10.1111/jhn.12582. 29984532

[ref94] Netzel M.; Strass G.; Janssen M.; Bitsch I.; Bitsch R. Bioactive anthocyanins detected in human urine after ingestion of blackcurrant juice. J. Environ. Pathol Toxicol Oncol. 2001, 20 (2), 89–95. 10.1615/JEnvironPatholToxicolOncol.v20.i2.20. 11394716

[ref95] Costello R.; Keane K. M.; Lee B. J.; Willems M. E. T.; Myers S. D.; Myers F.; Lewis N. A.; Blacker S. D. Plasma uptake of selected phenolic acids following New Zealand blackcurrant extract supplementation in humans. J. Diet Suppl. 2021, 0 (0), 1–16. 10.1080/19390211.2021.1914802. 33949254

[ref96] Quirós-Sauceda A. E.; Oliver Chen C. Y.; Blumberg J. B.; Astiazaran-Garcia H.; Wall-Medrano A.; González-Aguilar G. A. Processing ‘ataulfo’ mango into juice preserves the bioavailability and antioxidant capacity of its phenolic compounds. Nutrients 2017, 9 (10), 1082 10.3390/nu9101082. 28961171 PMC5691699

[ref97] Lutz M.; Castro E.; García L.; Henríquez C. Bioavailability of phenolic compounds in grape juice cv. Autumn Royal. CYTA - J. Food. 2014, 12 (1), 48–54. 10.1080/19476337.2013.793213.

[ref98] García-Muñoz C.; Hernández L.; Pérez A.; Vaillant F. Diversity of urinary excretion patterns of main ellagitannins’ colonic metabolites after ingestion of tropical highland blackberry (Rubus adenotrichus) juice. Food Res. Int. 2014, 55, 161–169. 10.1016/j.foodres.2013.10.049.

[ref99] Felgines C.; Talavera S.; Texier O.; Gil-Izquierdo A.; Lamaison J. L.; Remesy C. Blackberry anthocyanins are mainly recovered from urine as methylated and glucuronidated conjugates in humans. J. Agric. Food Chem. 2005, 53 (20), 7721–7727. 10.1021/jf051092k. 16190623

[ref100] Marques C.; Fernandes I.; Norberto S.; Sá C.; Teixeira D.; de Freitas V.; Mateus N.; Calhau C.; Faria A. Pharmacokinetics of blackberry anthocyanins consumed with or without ethanol: A randomized and crossover trial. Mol. Nutr Food Res. 2016, 60 (11), 2319–2330. 10.1002/mnfr.201600143. 27306520

[ref101] Kresty L. A.; Fromkes J. J.; Frankel W. L.; Hammond C. D.; Seeram N. P.; Baird M.; Stoner G. D. A phase I pilot study evaluating the beneficial effects of black raspberries in patients with Barrett’s esophagus. Oncotarget 2018, 9 (82), 35356–35372. 10.18632/oncotarget.10457. 30450163 PMC6219678

[ref102] Tian Q.; Giusti M. M.; Stoner G. D.; Schwartz S. J. Urinary excretion of black raspberry (Rubus occidentalis) anthocyanins and their metabolites. J. Agric. Food Chem. 2006, 54 (4), 1467–1472. 10.1021/jf052367z. 16478275

[ref103] Straßmann S.; Passon M.; Schieber A. Chemical hemisynthesis of sulfated cyanidin-3-o-glucoside and cyanidin metabolites. Molecules 2021, 26 (8), 2146 10.3390/molecules26082146. 33917913 PMC8068276

[ref104] Howard L. R.; Prior R. L.; Liyanage R.; Lay J. O. Processing and storage Effect on berry polyphenols: Challenges and implications for bioactive properties. J. Agric. Food Chem. 2012, 60 (27), 6678–6693. 10.1021/jf2046575. 22243517

[ref105] Mostafa H.; Meroño T.; Miñarro A.; Sánchez-Pla A.; Lanuza F.; Zamora-Ros R.; Rostgaard-Hansen A. L.; Estanyol-Torres N.; Andres-Lacueva C.; et al. Dietary Sources of Anthocyanins and Their Association with Metabolome Biomarkers and Cardiometabolic Risk Factors in an Observational Study. Nutrients. 2023, 15 (5), 1208 10.3390/nu15051208. 36904207 PMC10005166

[ref106] Spencer J. P. E.; Abd El Mohsen M. M.; Minihane A. M.; Mathers J. C. Biomarkers of the intake of dietary polyphenols: Strengths, limitations and application in nutrition research. Br. J. Nutr. 2008, 99 (1), 12–22. 10.1017/S0007114507798938. 17666146

[ref107] Wilson T.; Garcia-Perez I.; Posma J. M.; Lloyd A. J.; Chambers E. S.; Tailliart K.; Zubair H.; Beckmann M.; Draper J.; et al. Spot and Cumulative Urine Samples Are Suitable Replacements for 24-h Urine Collections for Objective Measures of Dietary Exposure in Adults Using Metabolite Biomarkers. J. Nutr. 2019, 149 (10), 1692–1700. 10.1093/jn/nxz138. 31240300

[ref108] Del Rio D.; Rodriguez-Mateos A.; Spencer J. P. E.; Tognolini M.; Borges G.; Crozier A. Dietary (poly)phenolics in human health: Structures, bioavailability, and evidence of protective effects against chronic diseases. Antioxidants Redox Signal. 2013, 18 (14), 1818–1892. 10.1089/ars.2012.4581. PMC3619154 22794138

[ref109] Marín L.; Miguélez E. M.; Villar C. J.; Lombó F. Bioavailability of dietary polyphenols and gut microbiota metabolism: Antimicrobial properties. Biomed Res. Int. 2015, 2015, 1 10.1155/2015/905215. PMC4352739 25802870

[ref110] Godos J.; Vitale M.; Micek A.; Ray S.; Martini D.; Del Rio D.; Riccardi G.; Galvano F.; Grosso G. Dietary polyphenol intake, blood pressure, and hypertension: A systematic review and meta-analysis of observational studies. Antioxidants. 2019, 8 (6), 1–21. 10.3390/antiox8060152. PMC6616647 31159186

[ref111] Liu H.; Garrett T. J.; Su Z.; Khoo C.; Gu L. UHPLC-Q-Orbitrap-HRMS-based global metabolomics reveal metabolome modifications in plasma of young women after cranberry juice consumption. J. Nutr Biochem. 2017, 45, 67–76. 10.1016/j.jnutbio.2017.03.007. 28433923

