# Supplementary material for: Biomarkers of Berry Intake: Systematic Review Update
Source: J Agric Food Chem. 2023 Jul 27;71(31):11789–805. doi: 10.1021/acs.jafc.3c01142 (PMC10416351; doi:10.1021/acs.jafc.3c01142)
Supplement: Supplementary file 1 — jf3c01142_si_001.pdf [file jf3c01142_si_001.pdf]

Supporting Information

Biomarkers of berry intake: Systematic review update

Hamza Mostafa[1,2], Alex Cheok[3], Tomás Meroño[1,2], Cristina Andres-Lacueva[1,2*], Ana Rodriguez-Mateos[3*]

[1] Biomarkers and Nutrimetabolomics Laboratory, Department of Nutrition, Food Sciences and Gastronomy, Nutrition and Food Safety Research Institute (INSA), Facultat de Farmàcia i Ciències de l'Alimentació, Universitat de Barcelona (UB), 08028 Barcelona, Spain.

[2] Centro de Investigación Biomédica en Red de Fragilidad y Envejecimiento Saludable (CIBERFES), Instituto de Salud Carlos III, Madrid, 28029, Spain.

[3] Department of Nutritional Sciences, School of Life Course and Population Sciences, Faculty of Life Sciences and Medicine, King's College London, 150 Stamford Street, SE1 9NH, London, UK.

[*]Corresponding author:

Cristina Andres-Lacueva. [*]Email: candres@ub.edu

Ana Rodriguez-Mateos. [*]Email: ana.rodriguez-mateos@kcl.ac.uk

Supplementary Table 1: Keywords used for the search in Ulaszewska M. et al. systematic review

Database / keywords	(Fruit name* OR botanical name), AND (urine or plasma or serum or excretion or blood) AND (human* OR men OR women OR patient* OR volunteer* OR participant*) AND (biomarker* OR marker* OR metabolite* OR biokinetics OR biotransformation OR pharmacokinetics OR bioavailability OR ADME) AND (intake OR meal OR diet OR ingestion OR administration OR consumption OR eating OR drink*)
PubMed	All fields
Web of Science	Topic
Scopus	Article Title/Abstract/Keywords
